# Fortifying angiogenic efficacy of conditioned media using phototoxic‐free blue light for wound healing

**DOI:** 10.1002/btm2.10462

**Published:** 2022-12-08

**Authors:** Sung‐Won Kim, Gwang‐Bum Im, Yeong Hwan Kim, Suk Ho Bhang

**Affiliations:** ^1^ School of Chemical Engineering, Sungkyunkwan University Suwon South Korea; ^2^ Present address: Department of Cardiac Surgery Boston Children's Hospital and Harvard Medical School Boston Massachusetts USA

**Keywords:** angiogenesis, bOLED, conditioned medium, phototoxicity, ROS

## Abstract

We used a blue organic light‐emitting diode (bOLED) to increase the paracrine factors secreted from human adipose‐derived stem cells (hADSCs) for producing conditioned medium (CM). Our results showed that while the bOLED irradiation promotes a mild‐dose reactive oxygen generation that enhances the angiogenic paracrine secretion of hADSCs, it does not induce phototoxicity. The bOLED enhances paracrine factors via a cell‐signaling mechanism involving hypoxia‐inducible factor 1 alpha. This study demonstrated that the CM resulting from bOLED treatment shows improved therapeutic effects on mouse wound‐healing models. This method contributes to overcoming the barriers to stem‐cell therapies, including the toxicity and low yields from other methods such as nanoparticles, synthetic polymers, and even cell‐derived vesicles.

## 1 INTRODUCTION

Stem cell therapy has been widely used in wound healing through paracrine factors secreted from transplanted stem cells^1^, ^2^ While adult stem cells show low side effects (e.g., immune response due to their immunomodulatory properties) and do not raise ethical issues,^3^, ^4^ low viability of human adipose‐derived stem cells (hADSCs) caused by harsh condition of damaged tissue remains as a problem that needs to be addressed.^5^ Although materials ranging from scaffolds to nanoparticles have been used to increase the therapeutic effects of stem cell therapy,^6^, ^7^, ^8^, ^9^, ^10^ abnormal immune responses attributed to low biodegradability and long‐term side effects of these materials limit their bio‐application.^11^, ^12^ Moreover, the potential risk of carcinogenesis in the administering of stem cells is believed to be an obstacle to their uses in clinical settings.^13^

To this end, conditioned medium (CM) promises to resolve the potential problem of transplant damage in stem cells.^14^, ^15^ The CM includes paracrine factors normally extracted from various cell cultures,^16^ such as cytokines, growth factors, hormones, and other soluble factors that regulate cellular functions through ligand‐receptor interactions via the cell signaling pathway. The application of CM ranges from osteogenesis, muscle regeneration, to ischemic disease.^17^, ^18^, ^19^ In particular, the angiogenic efficacy of CM harvested from adult stem cells has drawn much attention due to its robust wound healing properties.^20^, ^21^, ^22^, ^23^, ^24^ However, the low concentrations of therapeutic paracrine factors collected from normally cultured stem cells limit the widespread therapeutic usage.^14^ While numerous attempts have been made to increase the therapeutic effects of CM by introducing external materials (including growth factors, hydrogels, and even designed biocompatible nanomaterials) to the stem cells, the high cost, process complexity, and nanomaterial contamination risks in CM still challenge its application.^25^, ^26^, ^27^

Recently, the use of external stresses, such as light, acoustics, and shear stress instead of introducing external materials has gained much attention.^11^, ^28^, ^29^ Among the external stimuli, light energy is widely accepted as a promising tool for wound healing.^30^ In particular, red visible light (wavelength ~ 650 nm) applied to wound areas in previous research^30^ has been shown to enhance the angiogenic efficacy of stem cells, thereby showing stimulated therapeutic efficacy.^31^ Furthermore, blue visible light (~420 nm) with high‐density energy, has attracted attention as a promising tool for use in various fields, including soft robotics and cell sheet engineering.^32^, ^33^ However, to date, only a few studies have focused on blue visible light in therapeutic‐applications because of its phototoxic effect on the cells, attributed to its reactive oxygen species (ROS)‐generating property.^34^ When cells are exposed, cytochrome c in mitochondria absorb the blue light, which disrupts electron transport and causes ROS generation.^35^, ^36^ The few existing studies include attempts to apply blue light to increase osteogenic^37^ and angiogenic properties.^38^ Blue light stem cell therapies, however, still need to be thoroughly explored in terms of cellular mechanisms and in vivo therapeutic efficacy before being used in clinical settings.

In this study, we used a blue organic light‐emitting diode (bOLED) to increase the paracrine factors secreted from hADSCs, with controlled irradiation to reduce phototoxicity. Our findings elucidated the mechanism underlying redox signaling induced by mild ROS generation in which bOLED irradiation enhances the angiogenic paracrine secretion of hADSCs. We demonstrated the improvement of in vivo wound healing efficacy by injecting the CM extracted from bOLED light‐treated hADSCs into the wound lesions of mice. Optimized condition of our bOLED‐based CM therapy has a potential to replace the current clinical settings of cell therapy with advantages on cell function and CM manufacturing time and cost.

## 2 RESULTS

### 2.1 Characteristics of bOLED and culture condition

**FIGURE 1:**
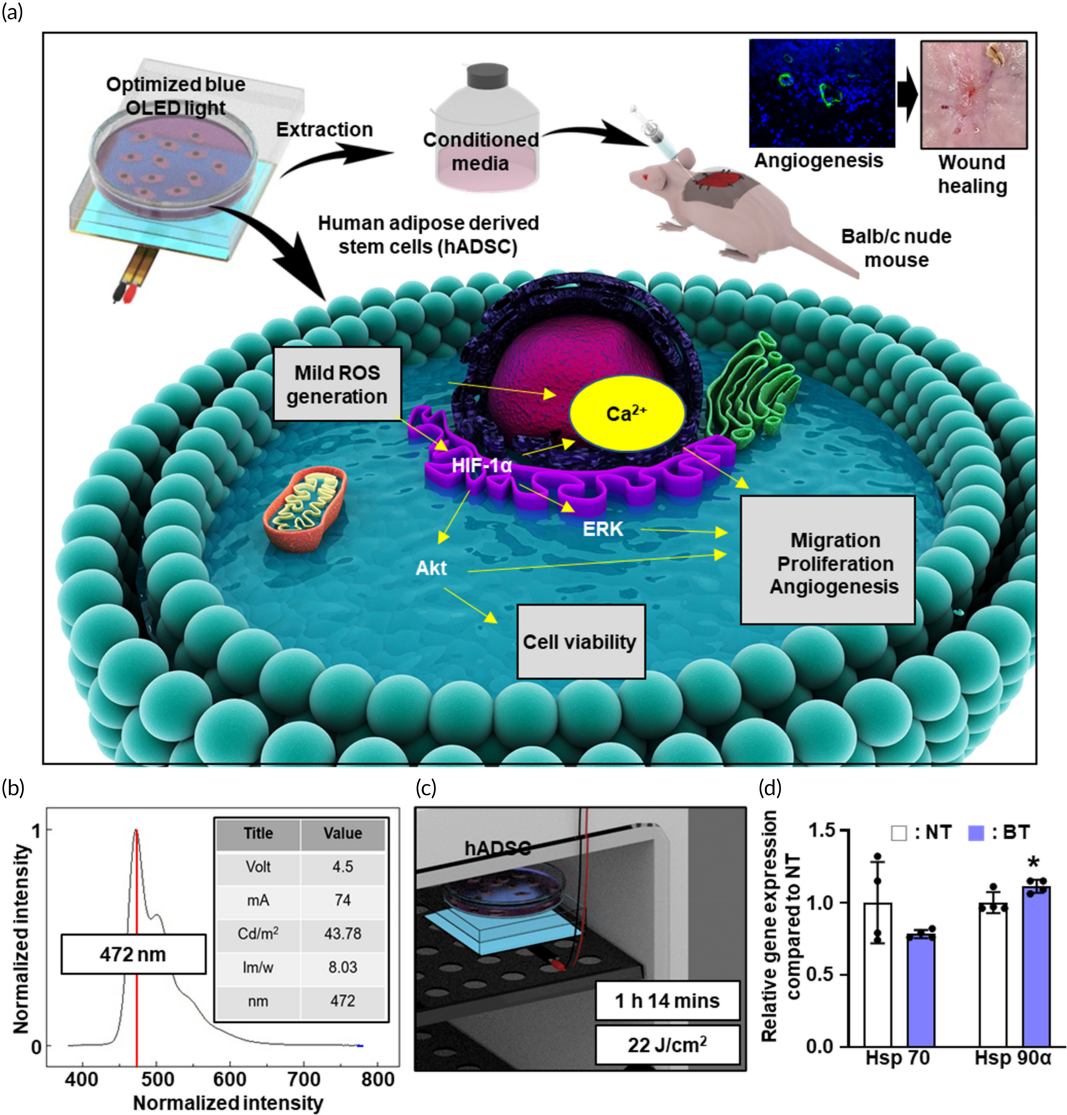
Schematic illustration of the study and characterization of blue organic light‐emitting diode (bOLED). (a) Study overview including molecular mechanisms. (b) Characteristics of bOLED used in this study. Profile of wavelength including peak wavelength of bOLED. (c) Description of bOLED setting and human adipose‐derived stem cells (hADSC) culture in the incubator. (d) Gene expression related with heat induced cellular damage (Data are presented as means ± SDs. *p < 0.05 versus NT).

In this study, we optimized the blue OLED light irradiation for hADSC culture to induce a mild‐dose ROS generation (Figure 1a). We selected a bOLED with stable fixed values such as luminance of 43.78 Cd/m^2^, power efficiency of 8.03 lm/w, and peak wavelength of 472 nm wavelength when 4.5 V and 74 mA were applied. Figure 1b shows that the bOLED has a broad light spectrum biased towards green and red regions with a peak at 472 nm. To transfer the optimized energy to hADSCs, we irradiated the bOLED for 1 h 14 min, which had an optimized fluence of 22 J/cm^2^, as in our previous study.^39^ (Figure 1c). To determine whether our light system induces heat generation, we evaluated the expression of genes related to heat, such as heat shock protein (Hsp) 70 and Hsp 90α. While we found no significant difference between the two groups in Hsp 70 expression, a slight increase was observed in Hsp 90α expression in bOLED‐treated hADSCs compared to hADSCs without bOLED treatment (Figure 1d).

### 2.2 In vitro phototoxicity of hADSCs

**FIGURE 2:**
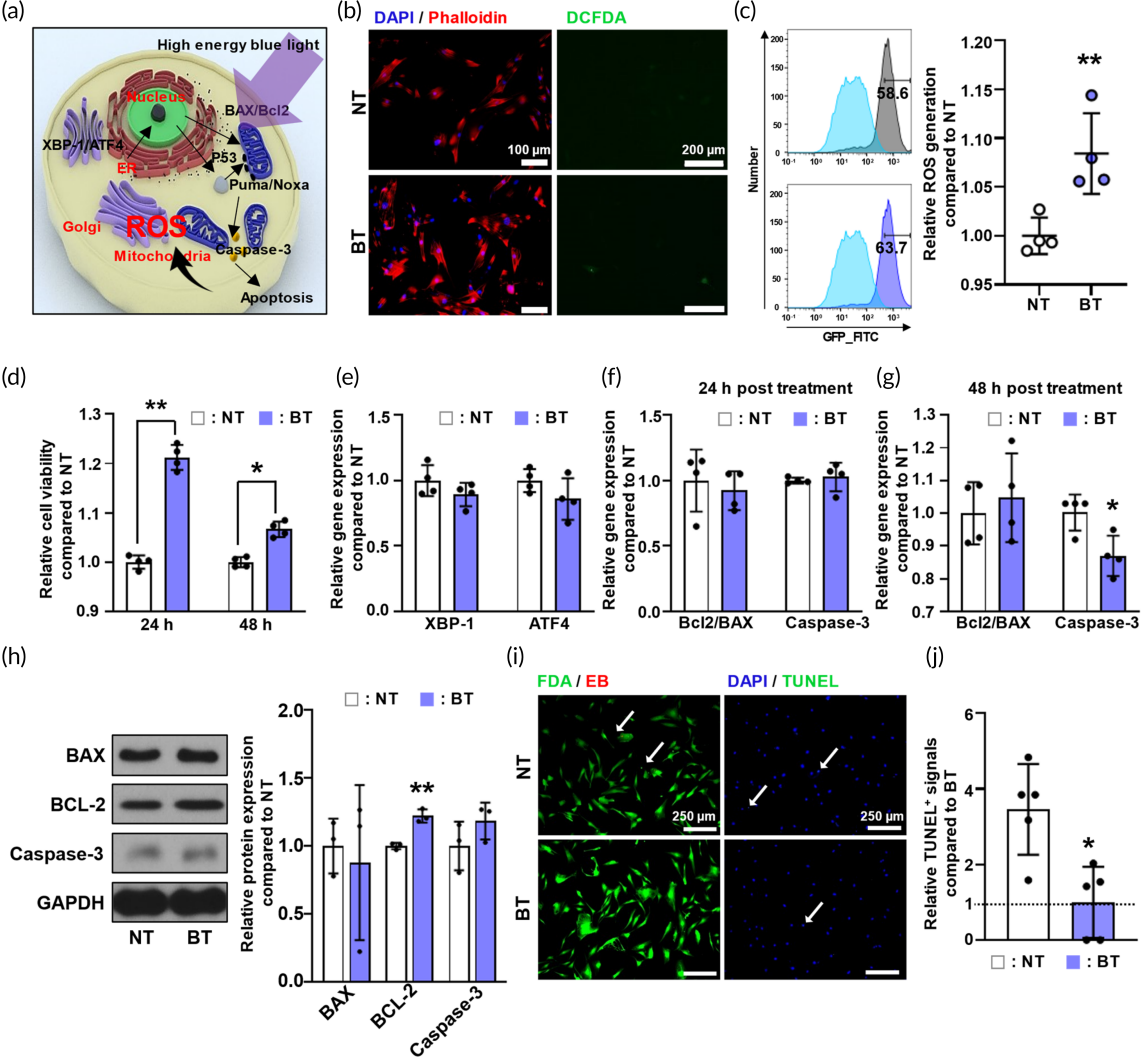
Apoptotic activity of human adipose‐derived stem cells (hADSCs) irradiated with blue organic light‐emitting diode (bOLED). (a) Schematic diagram of hADSC showing phototoxicity irradiated after high energy blue light. (b) Co‐staining of hADSC with phalloidin and DAPI, which stain cytosol with red and nuclei with blue (Left). DCFDA (2′,7′‐dichlorofluorescin diacetate) staining for detecting reactive oxygen species (ROS) generation of hADSC. (c) Quantification of ROS generation evaluated by FACS analysis (*n* = 4). (d) Relative cell number of the hADSCs at 24 and 48 h after bOLED irradiation. (e) ER stress of hADSC irradiated after 24 h. Apoptotic gene expression of the hADSCs irradiated after (f) 24 h and (g) 48 h. (h) Western blot analysis of apoptosis‐related protein expression in hADSCs irradiated after 24 h (*n* = 3) (i) Apoptotic cells stained with FDA/EB staining and TUNEL staining at 48 h with or without bOLED irradiation, which stain each of apoptotic cell with red and green. (j) Quantification of the number of TUNEL signals in each group. Data are presented as means ± SDs. *p < 0.05, **p < 0.01 versus NT.

High‐energy blue light is known to induce phototoxicity in cells. When cytochromes of the respiratory mitochondria absorb blue light, ROS is generated by disruption of electron transport (Figure 2a).^32^, ^33^, ^40^ High‐energy blue light irradiation causes both endoplasmic reticulum (ER) stress and intracellular ROS levels in cells to rise, thereby increasing cell death‐related genes to induce apoptosis.^41^, ^42^ However, we found that when OLED light with optimized light energy was used for irradiation, the actin filaments of hADSCs, which are cleaved during apoptosis,^43^, ^44^ underwent no change compared with samples that were not irradiated with bOLED light (Figure 2b). To investigate whether ROS generation was increased in blue light‐irradiated hADSCs, we performed 2′,7′‐dichlorofluorescin diacetate (DCFDA) staining. As shown in Figure 2b, the bOLED‐light irradiation increased ROS generation in hADSCs. While the fluorescein signals in the FITC channel were rarely found in the normally cultured two‐dimensional (2D) group (NT), they were observed as green signals in the bOLED‐treated hADSCs (BT) group. Further flowcytometry (FACS) analysis confirmed the higher expression of DCFDA in BT compared to NT. BT group showed about 10% higher ROS generation than that of the NT group (Figure 2c). In addition, bOLED‐light irradiation increased cell proliferation in the BT group compared to that in the NT group for both 24 and 48 h after irradiation (Figure 2d). We further compared the effects of bOLED‐irradiation on the gene expressions of both *XBP1* and *ATF4* and found no difference between the NT and the BT groups (Figure 2e). This indicates that the unfolded protein response activated by ER‐stress to maintain ER homeostasis did not occur following bOLED‐irradiation, as would be expected in ER stress‐mediated apoptosis.^45^, ^46^ In addition, we compared the effects of bOLED‐irradiation on both the cell survival susceptibility in terms of BCL2/BAX ratios, and on the cell‐death marker CASPASE‐3. Our results indicated that while no change was observed 24 h after bOLED irradiation (Figure 2f), a significant decrease in *CASPASE‐3* gene expression levels by 85% was observed in the BT group compared to the NT group 48 h after bOLED irradiation (Figure 2g). The western blot analysis showed no phototoxic effect of bOLED irradiation on hADSCs. (Figure 2h). Despite no significant differences were confirmed in Caspase‐3 and BAX expression in BT, one of the representative anti‐apoptotic markers, BCL‐2 was significantly increased about 1.2 times enhanced compared to NT (Figure 2h). In addition, the bOLED treatment decreased the ethidium bromide (EB)‐positive signal, which is known to diffuse across an apoptotic or necrotic cell membrane (left panels of Figure 2i). Furthermore, the terminal deoxynucleotide transferase‐mediated deoxyuridine triphosphate nick end labeling (TUNEL) signals, associated with DNA fragmentation in the last phase of apoptosis, were rarely observed for both NT and BT (right panels of Figure 2i). TUNEL signals of NT are about two times larger than that of BT (Figure 2j). From the above results, we concluded that the bOLED light increased both the proliferation and the viability, presumably by increasing redox signaling induced by mild‐dose ROS generation.

### 2.3 In vitro mechanism of ROS‐related angiogenic expression and biomodulation

**FIGURE 3:**
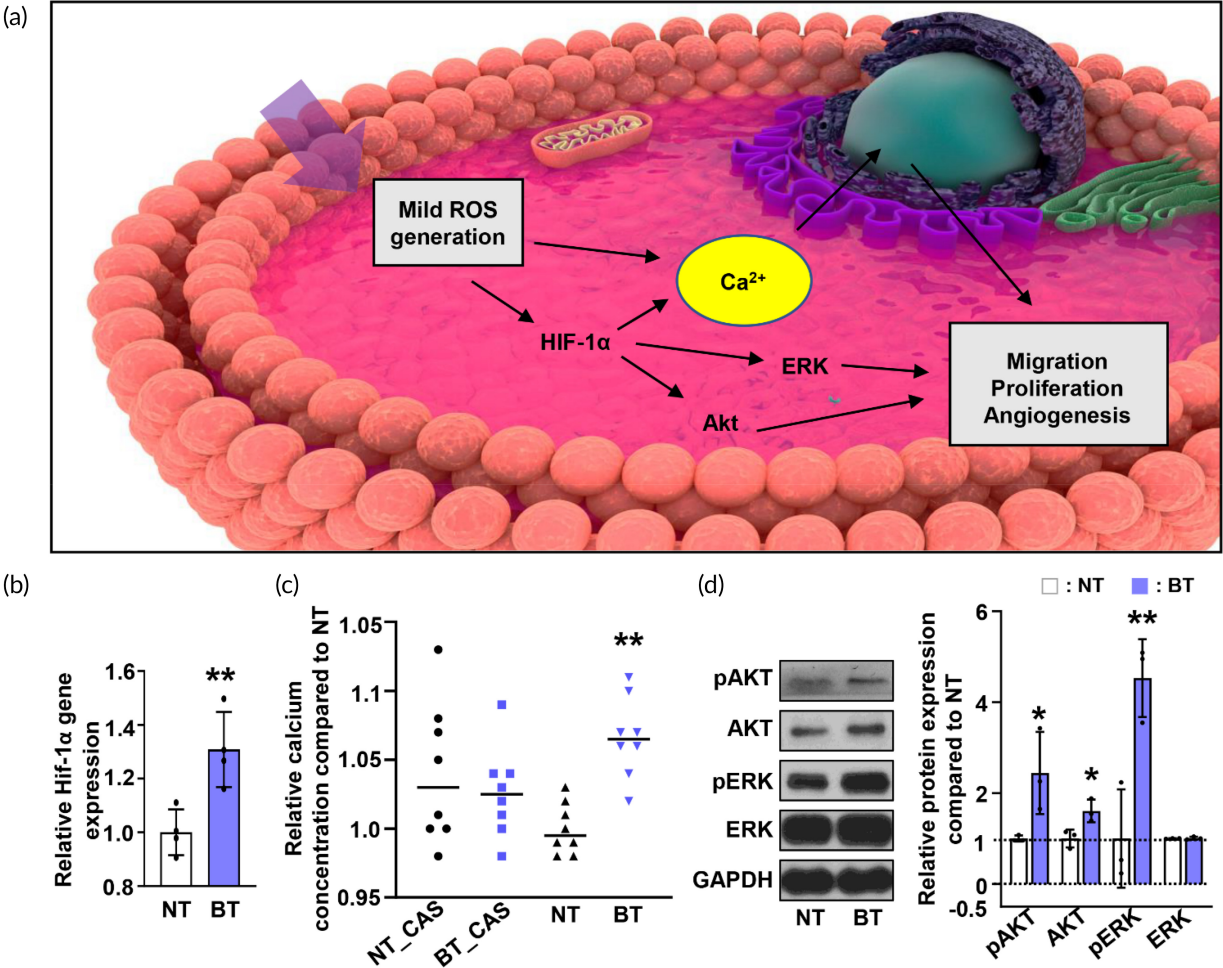
(a) Schematic illustration of redox signaling induced by mild reactive oxygen species (ROS) generation. (b) quantitative polymerase chain reaction (qRT‐PCR) of the HIF‐1α (*n* = 4). (c) Comparison of relative calcium concentration in HIF‐1α inhibitor pre‐treated hADSCs irradiated with blue organic light‐emitting diode (bOLED) light (*n* = 8). (d) Protein expression of ERK and AKT in hADSC irradiated with bOLED light. *p < 0.05, **p < 0.01 versus NT.

Figure 3a illustrates our hypothesis that bOLED light enhances redox signaling by mild‐dose ROS generation, which simulates both the HIF‐1α and the calcium cascade related to cell proliferation, viability, and angiogenic properties. We used quantitative polymerase chain reaction (qRT‐PCR) to confirm that bOLED light increases *HIF‐1α* gene expression by 30%, as shown in Figure 3b. According to the previous research, calcium is known to be controlled by HIF‐1α expression and increase angiogenic efficacy.^47^, ^48^, ^49^ Next, we demonstrated that bOLED light increases the calcium concentration via HIF‐1α upregulation. We treated hADSCs with an HIF‐1α inhibitor (CAY10585) immediately before irradiation with bOLED light to investigate whether HIF‐1α expression influences calcium deposition. As shown in Figure 3c, no difference was observed between CAY10585 pre‐treated with either bOLED‐treated (BT_CAY) or nontreated hADSCs (NT_CAY). As opposed to the CAY10585‐treated hADSC, the amount of calcium detected in the BT group was significantly higher than that in the NT group (Figure 3c). Previous studies demonstrating the endothelial Ca^2+^ signaling effects on the upregulation of angiogenesis and vasculogenesis support our observations.^49^ The western blot analysis further indicated that bOLED light stimulates the cell signaling involved in AKT and ERK mediated pathways—which are known to promote cell proliferation, viability, and angiogenic properties (Figure 3d).^50^, ^51^, ^52^ As shown in Figure 3d, the amount of the phosphorylated AKT and ERK in BT was 2.4 and 4.5 times larger than those of NT.

**FIGURE 4:**
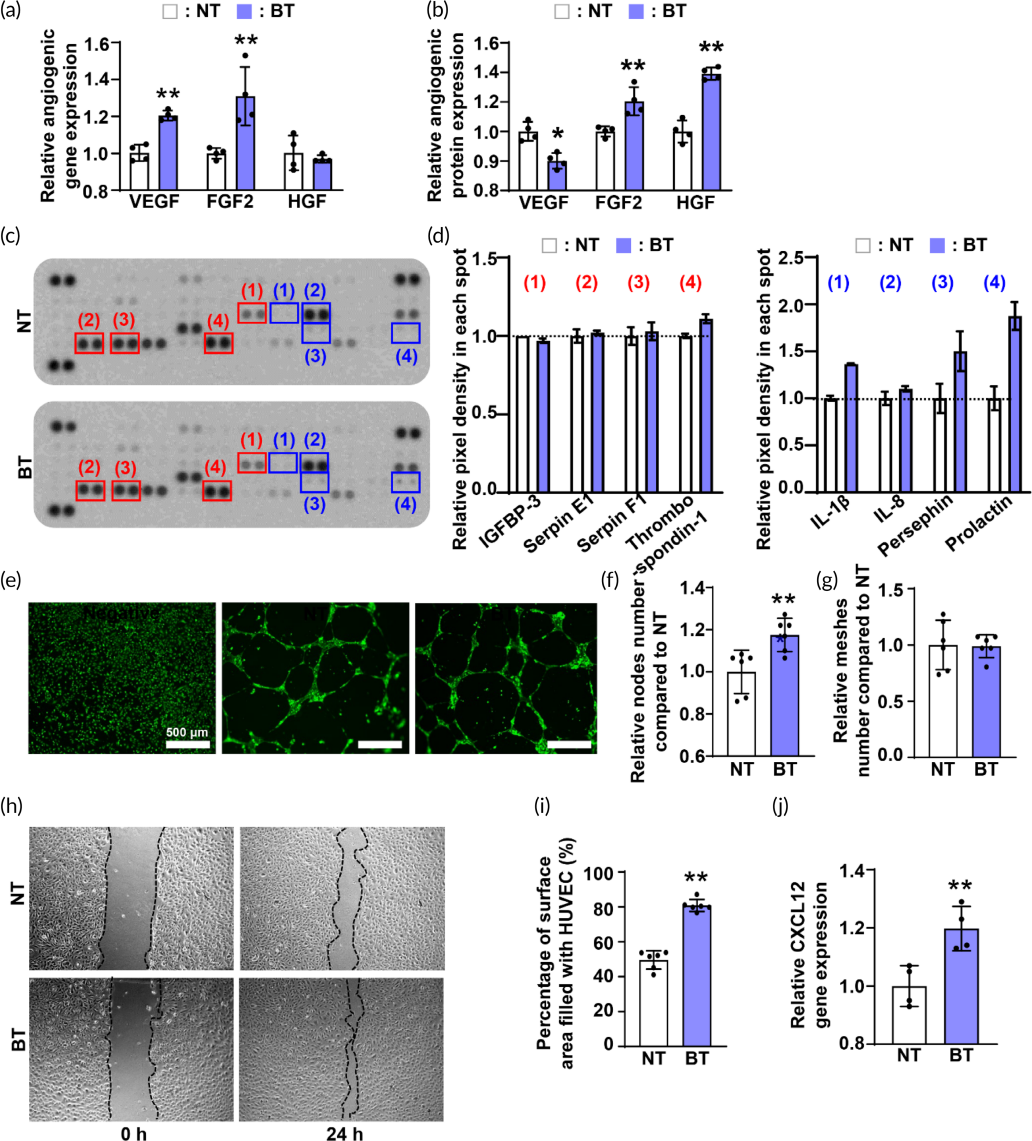
Upregulated angiogenic capacity of human adipose‐derived stem cells (hADSCs), (a) VEGF, HGF, and FGF2 gene expression compared to NT at 48 h after irradiation. (b) Angiogenic protein secreted from hADSCs with blue organic light‐emitting diode (bOLED) irradiation (BT) in CM; VEGF, HGF, and FGF2 evaluated via ELISA assay (*n* = 4). (c) Angiogenesis array and (d) its quantification showing the angiogenesis‐related chemokines and cytokines. (e) Images of tube formation after treating CM collected from BT, CM, and its quantification via counting the number of (f) meshes and (g) nodes. (h) Migration efficacy of human umbilical vein endothelial cells treated with CM analyzed by wound scratch assay and (i) its quantification. (j) *CXCL12* gene expression analyzed with qRT‐PCR. Data are presented as means ± SDs. *p < 0.05, **p < 0.01 versus NT.

To examine whether angiogenic properties of hADSCs were increased as we had hypothesized, we investigated a series of genes (Figure 4a) and proteins (Figure 4b) related to angiogenesis. Figure 4a showed that bOLED irradiation enhances both the Vascular endothelial growth factor (*VEGF)* and Fibroblast growth factor 2 (*FGF2)* gene expressions in hADSCs. Furthermore, the secreted FGF2 and hepatocyte growth factor (HGF) in the BT group were about 1.2 and 1.4 times higher, respectively, than those of NT group (Figure 4b). For HGF, the protein secretion in the BT group was upregulated in Figure 4b, as opposed to a 10% decrease in *HGF* gene expression in Figure 4a. For VEGF, although the gene expression in the BT group was higher than that in the NT group, the secreted amount was slightly decreased by ELISA (Figure 4b). To clarify the angiogenic profiles of hADSC irradiated by bOLED light, we performed an angiogenesis array using the CM extracted from each group (Figure 4c). The results shown in Figure 4d confirm the enhancement in angiogenesis‐related chemokines and cytokines, such as serpin E1, serpin F1, thrombospondin‐1, interleukin (IL)‐1 beta, IL‐8, persephin, and prolactin. Among the various factors, only IGFBP‐3, which is associated with both pro‐ and anti‐angiogenesis, decreased. To assess the in vitro angiogenic properties of the CM extracted from hADSCs after bOLED irradiation, we investigated the tube formation properties of the human umbilical vein endothelial cells (HUVECs) in Figure 4e–g. The negative group, which was treated with fresh Dulbecco's modified Eagle's medium (DMEM), formed the same number of meshes as the BT group (Figure 4g). However, the number of tubular nodes, a criterion of enhancement in tube formation, was remarkably increased up to 20% in the BT group (Figure 4f). To determine the migration properties of bOLED light‐treated hADSCs, we performed a wound scratch assay. Figures 4h,i show that the HUVECs in the CM of the BT group increased in cell migration compared to that in the CM of the NT group. Furthermore, Figure 4i also shows the HUVEC migration area of the BT group was quantitatively enhanced by more than 30% compared to that of the NT group. Similar to the enhanced migration of the HUVECs in the BT group, the gene expression level of *CXCL12*, also known as SDF1,^53^ was significantly increased in the BT CM‐treated HUVECs (Figure 4j).

### 2.4 Angiogenesis improved by CM in an in vivo mouse wound model

**FIGURE 5:**
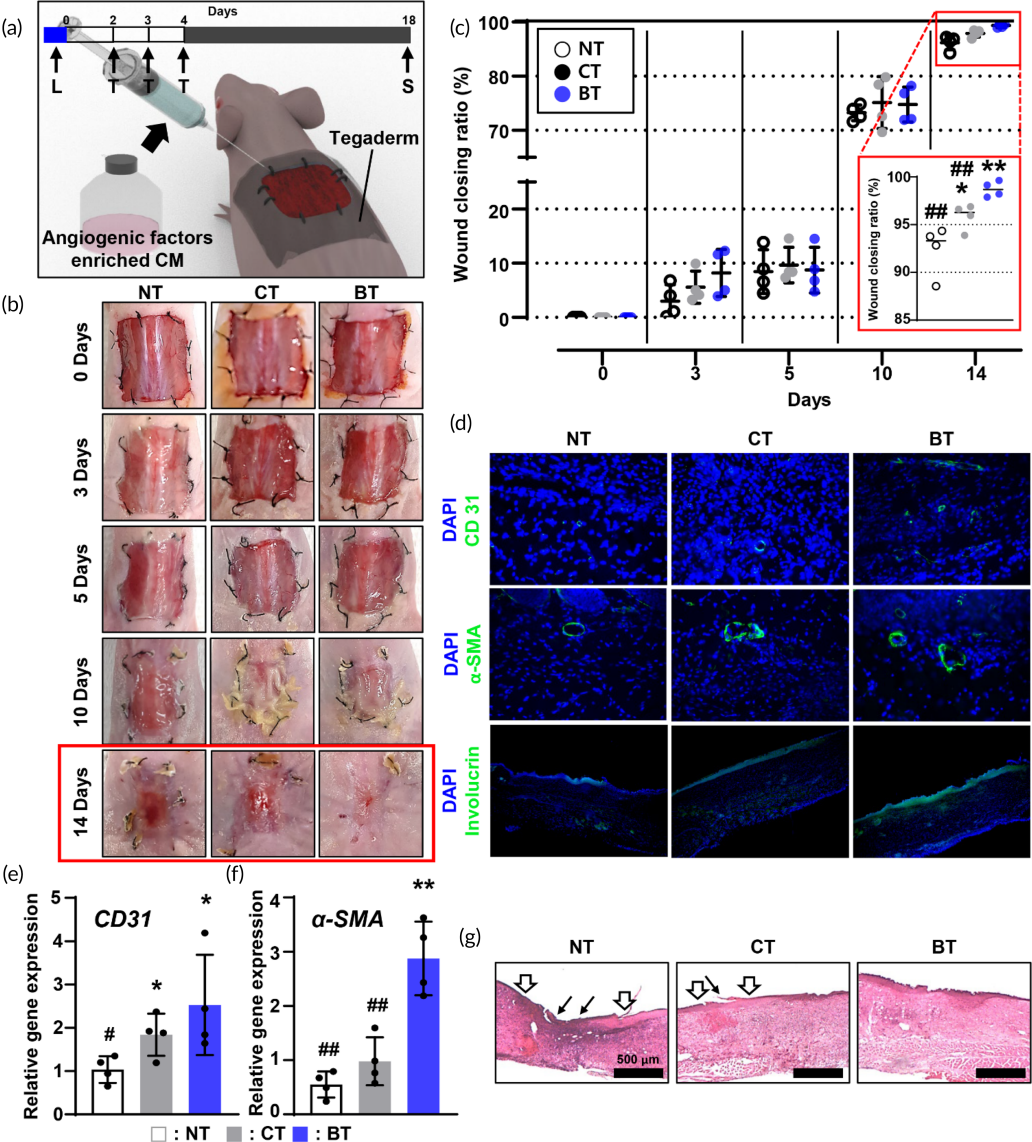
Wound closure improved in vivo skin wound mouse model by injecting blue organic light‐emitting diode (bOLED)‐treated conditioned medium (CM) injection. (a) Schematic diagram of in vivo test including the time point of each treatment. L indicated the time of light irradiation and T indicated the time of CM treatment (b) Representative photographs of no treatment (NT), injecting CM derived from human adipose‐derived stem cells (hADSCs) without bOLED treatment (CT), and injecting CM derived from hADSCs with bOLED treatment (BT) groups on days 0, 3, 5, 10, and 14 after CM injection. (c) Wound closing ratio of each group on Days 0, 3, 5, 10, and 14 after various treatments (*n* = 4). Red box showed wound closing ratio result of each group in 14 days after treatments. Immunofluorescent staining of DAPI and (d) CD31, smooth muscle α‐actin (α‐SMA), and involucrin in skin wound regions at 14 days after treatments. Quantitative polymerase chain reaction (qRT‐PCR) results showing (e) *CD3*1 and (f) *α‐SMA* wound sites at 14 days after treatments. (g) H&E staining of wound healing regions at 14 days after treatments in each group. Black and white arrows indicated defects of re‐epithelization. Data are presented as means ± SDs (*n* = 4). **p < 0.01, *p < 0.05 versus OT group, *##p* < 0.01 versus each BCT group.

To assess the therapeutic effects, we injected the above CMs into skin wounds on BALB/c nude mice models on Days 2–4 (Figure 5a). Figure 5b compares the healing process of the group untreated group (NT), with those that were treated with the CM extracted from non‐bOLED‐irradiated hADSCs (CT) and with the CM extracted from bOLED‐irradiated hADSCs (BT). As this figure shows, the skin wound closure was remarkably enhanced in the BT group (98.7%) compared to that in the NT (92.4%) and CT (95,8%) groups. In particular, most of the wound region appeared to be closed 14 days after BT (red box). Similar to the representative images of wound‐healing profiles, the wound closing ratio increased significantly in the BT group compared to the other groups (Figure 5c). The skin wound restoration was also observed in the CT group compared to the OT group, but the extent was lower than that in the BT group (Figure 5c). Figure 5d shows the representative vascular markers, including CD31 and Alpha‐smooth muscle actin (α‐SMA) exhibiting stronger fluorescent signals in the BT group compared to the other groups. Involucrin, a marker of epidermal differentiation in skin substitutes, was also increased in the BT group compared to both the OT and CT groups (Figure 5d). The total amount of *CD31* and *α‐SMA* gene expression levels in the wound region were significantly upregulated in the BT group compared to those in the OT and CT groups (Figure 5e, f). As shown in the H&E staining results (Figure 5g), the BT group showed enhanced re‐epithelization with increased thickness of the epidermis.

## 3 DISCUSSION

Recently, tremendous efforts incorporating external materials into cells have been made to overcome the limited therapeutic effect of conventional cell therapy. However, although the transplanted cell survival rate and growth factor secretion were increased with incorporated materials, side effects, such as immune response limit widespread application. To this end, CM therapies have drawn attention as an alternative tool that can avoid immune side effects. Numerous attempts have been made to increase the concentrations of growth factors by stimulating cell biomodulation. While light is a promising tool enhancing cell biomodulation, blue wavelength light has not been fully applied because of its ROS‐generating property, which can induce cell apoptosis. In this context, we have used low‐energy‐density, low heat‐generating blue wavelength OLED light to generate mild‐dose ROS in hADSCs—inducing redox signaling to increase angiogenic growth factor secretion, while avoiding phototoxicity.

Since both mitochondria and NOX4 generate excessive amounts of ROS that can cause cellular damage when irradiated with high‐energy blue light, we irradiated relatively low‐energy blue light using OLEDs to induce redox signaling but without phototoxicity. Normally, OLEDs have low heat‐generating properties, which are suitable for excluding heat‐induced cellular damage during cell culture. We first confirmed that hADSCs treated with bOLED at 22 J/cm^2^ do not show a substantial heating effect caused by the light source as compared to that of the NT group. A slight ROS increase was observed with DCFDA staining in the BT group, but apoptotic activity was not observed in the BT group compared to that in the NT group due to relatively low ROS generation.

As mild‐dose ROS generation induces redox signaling through HIF‐1α upregulation, we confirmed that HIF‐1α expression affects the calcium concentration in the BT group, which is involved in various biological modulations, such as cell proliferation and migration. Phosphorylation of both ERK and AKT signaling pathways in the BT group was further demonstrated by western blotting, potentially affecting the cell viability and angiogenic properties of hADSCs. We concluded that HIF‐1α upregulation by means of redox signaling induced by mild ROS generation increases cell viability and furthers angiogenic properties by stimulating the AKT and ERK pathways.

Thereafter, we confirmed that more angiogenic growth factors, including HGF and FGF2, were secreted into the culture medium when irradiated with bOLED light compared to CM retrieved from hADSCs without bOLED treatment. The CM from the BT group further showed enhanced tube formation and migration ability of HUVECs compared to those of the NT group due to increased angiogenic growth factors. The angiogenesis array further demonstrated that bOLED light not only increased the pro‐angiogenic factors but also restricted anti‐angiogenic factors, which consolidated the fact that cytokines from BT increased vascularization.

Finally, we confirmed *the* in vivo wound healing effect by injecting CM from bOLED light‐treated hADSCs into the wound area. Apart from faster wound contraction, re‐epithelialization was found to increase in the BT group, consistent with the results of the in vitro test. The better vascularization of BT further demonstrated the therapeutic effect of bOLED light on hADSCs in our study.

## 4 CONCLUSION

In this study, we present an effective method to improve wound healing with a CM treatment that can avoid the problems of conventional stem cell therapy. In addition, unlike conventional CM therapies, the CM obtained here using bOLED is capable of treating hADSCs with significantly higher amounts of angiogenic growth factors without phototoxicity. Although the detailed mechanism of blue light in stem cell therapy needs to be elucidated in future studies, we have introduced a method for increasing the angiogenic effects of CM using bOLEDs to target hADSCs.

## 5 MATERIAL AND METHODS

### 5.1 Blue organic light‐emitting diode panel

A bOLED panel was obtained from the KANEKA Corporation (Tokyo, Japan). The OLED panel was in the incubator at 37°C with 5% CO_2_ saturation; wrapped in plastic bags to protect the OLED panel from humid conditions during incubation (95% humidity); and connected to a regulated direct‐current power supply that is installed on the exterior top of the incubator. (ODA Technologies, Incheon, Republic of Korea). The bOLED panel was placed underneath the cell‐culture plate (Figure 1c); the panel size was 8 cm × 8 cm—large enough to cover the cell‐culture plates.

### 5.2 Cell culture

Human adipose‐derived stem cells were purchased from Lonza (Basel, Switzerland) and cultured in DMEM (Gibco BRL, Gaithersburg, MD, USA) supplemented with 10% (v/v) fetal bovine serum (Gibco BRL) and 1% (v/v) penicillin/streptomycin (Gibco BRL). The cells were incubated at 37°C and 5% CO_2_ saturation. The medium was changed every 2 days. Cells within seven passages were used in the experiments. For CM extraction, hADSCs were treated with Cu‐AMN (Cu‐AMN CM) or received no treatment (NT CM). For the protein assay, NT CM or Cu‐AMN CM was extracted 1 day after treatment. The HUVECs were purchased from PromoCell (Heidelberg, Germany), and were cultured in endothelial cell media/media 2 (PromoCell) supplemented with Growth Medium 2 SupplementMix (PromoCell), and incubated at 37°C and 5% CO_2_ saturation. The medium was changed every 2 days.

### 5.3 Irradiation of bOLED light

Before irradiating hADSCs with blue light, the culture medium was replaced with a serum‐free culture medium following phosphate bovine saline (PBS, Gibco BRL, NY, USA). Thereafter, the bOLED light was irradiated for 1 h 14 min, and a total of 22 J/cm^2^ of light energy was transferred to the cultured hADSCs.

### 5.4 Measurement of cell viability of hADSCs

The cell viability was evaluated using a cell counting kit (CCK‐8) purchased from Dojindo Molecular Technologies, Inc. (Kumamoto, Japan). The CCK‐8 assay can measure the amount of formazan dye that is reduced by intracellular dehydrogenase activity, as the number of living cells is proportional to the amount of formazan dye. Briefly, hADSCs (1.5 × 10^4^ cells/well) were cultured in 24‐well plates. Before bOLED irradiation, the hADSCs were rinsed once with PBS. After replenishing the wells with fresh medium without serum, bOLED light was shone onto the hADSCs. The hADSCs were then cultured for an additional 24 h. Thereafter, CCK‐8 solution was added to each well and incubated for 2 h. The absorbance was measured at 450 nm using a microplate reader (Tecan, Männedorf, Switzerland). The number of cells was calculated as the percentage of viable cells relative to that of normal cells without bOLED treatment (*n* = 4).

### 5.5 TUNEL assay

After replenishing the wells with serum‐free medium, hADSCs were irradiated with or without bOLED light for 1 h and 14 min at 22 J/cm^2^. The hADSCs were then cultured for an additional 48 h. After rinsing the cells with PBS, the TUNEL assay was performed with an ApopTag® Fluorescent In‐Situ Apoptosis Detection Kit (Millipore, Bedford, MA, USA) to examine the apoptotic activity of hADSCs. After 4′,6‐diamidino‐2‐phenylindole (DAPI) (Vector Laboratories, Burlingame, CA, USA) staining, TUNEL‐positive fluorescence was measured with a fluorescence microscope (IX71 inverted microscope; Olympus, Tokyo, Japan).

### 5.6 Cell migration assay

The hADSCs were grown to confluence in 6‐well plates. The hADSCs were then replenished with endothelial cell growth medium‐2 (EGM‐2, Lonza Bioscience, Basel, Switzerland) and irradiated with or without bOLED light for 1 h and 14 min at 22 J/cm^2^. A straight scratch was made on the layer of hADSCs using a P1000 pipette tip. After incubating for 0 and 24 h, the gap width of the scratch re‐population was measured and compared to the initial gap size at 0 h. Using Adobe Photoshop CC (Adobe Systems, CA, USA), the size of the denuded area was determined at each time point from digital images.

### 5.7 Quantitative polymerase chain reaction

The qRT‐PCR was used to quantify relative gene expression levels of human‐specific *Hsp70, HSP90α, XBP1, ATF4, Bcl‐2, BAX, Caspase‐3, CXCL12, HIF‐1α, VEGF, FGF2, HGF*, mouse *β‐actin*, mouse *CD31*, and mouse *α‐SMA*. The primers for human genes were used for in vitro hADSC analyses. The primers for mouse genes were used for in vivo mouse skinwound tissue analyses. The total ribonucleic acids (RNAs) were extracted from samples using 1 ml TRIzol reagent (Life Technologies, Inc., Carlsbad, CA, USA) and 200 μl chloroform. The lysed samples were centrifuged at 12,000 rpm for 10 min at 4°C. The RNA pellets were washed with 75% (v/v) ethanol in water and dried. After drying, the samples were dissolved in RNase‐free water. For qRT‐PCR, a SsoAdvanceed Universal SYBR Green Supermix kit (Bio‐Rad, Hercules, CA, USA) and a CFX Connect™ real‐time PCR detection system (Bio‐Rad) were used.

### 5.8 Western blot analysis

The hADSCs were collected and lysed in a radioimmunoprecipitation assay buffer (Rockland Immunochemicals Inc., Limerick, PA, USA). After centrifugation at 10,000×*g* for 10 min, the supernatant was prepared as a protein extract. The protein concentrations were determined using a bicinchoninic acid assay (Pierce Biotechnology, Rockford, IL, USA). The same proteins from each sample were mixed with a sample buffer, loaded, and subjected to sodium dodecyl sulfate polyacrylamide gel electrophoresis (SDS‐PAGE) using a 10% (v/v) resolving gel. The proteins separated by SDS‐PAGE were transferred to immune‐blot polyvinylidene fluoride membranes (Bio‐Rad) and probed with antibodies against GAPDH (Abcam, ab9485, 1:1000, Cambridge, UK), BAX (Cell Signaling Technology, MA, USA, #5023, 1:1000), Bcl2 (Abcam, ab182858, 1:2000), Caspase‐3 (Cell Signaling Technology, $9662, 1:1000), ERK (Cell Signaling Technology, # 9101, 1:1000), p‐ERK (Cell Signaling Technology, #9102, 1:1000), AKT (Cell Signaling Technology, #4691, 1:1000), and pAKT (Cell Signaling Technology, #4060, 1:500) at 4°C overnight. The membranes were then incubated with horseradish peroxidase‐conjugated secondary antibody (R&D Systems, HAF008 for GAPDH, HAF017 for ERK, Caspase‐3, AKT, pAKT, BAX, Bcl2 1:1000, Minneapolis, MN, USA) for 1 h at room temperature. The blots were then developed in a dark room. The luminescence was recorded on an x‐ray blue film (Agfa HealthCare NV, Mortsel, Belgium). The bands were imaged using Photoshop CC.

### 5.9 Human angiogenesis and cytokine array

Human angiogenesis (ARY007, R&D Systems, Minneapolis, MN, USA) was conducted using the manufacturer's protocol.

### 5.10 Wound treatment

Eight‐week‐old female athymic mice (20–25 g body weight, Orient, Seoul, Korea) were anesthetized using 200 μl xylazine (20 mg/kg) and ketamine (100 mg/kg) diluted in normal saline solution. A square‐shaped (4 cm^2^) dorsal skin area was surgically removed. After skin removal, eight 6–0 sutures (AILEE Co., Busan, Korea) were placed at the border of each wound to prevent wound collapse due to skin contracture. Immediately after skin wound modeling, the mice were subdivided into three groups: OT (no treatment, wounds covered with only commercial skin dressing) (Tegaderm, 3 M Healthcare, St. Paul, MN, USA), CT (wounds injected with CM extracted from hADSC + Tegaderm), and BT (wounds injected with CM extracted from bOLED‐irradiated hADSC + Tegaderm) groups. In every group, 200 μl was injected into the wound area. The OT group served as the control group. All animals received care according to the Guidelines for the Care and Use of Laboratory Animals of Sungkyunkwan University (SKKUIACUC2020‐01‐12‐1, January 2020).

### 5.11 Histology

The skin tissue specimens were retrieved 14 days post‐treatment and fixed with a 4% formaldehyde solution. The samples were embedded in optimum cutting temperature (OCT) compound (SciGen Scientific, Gardenas, USA) and frozen to yield 10 μm sections. Hematoxylin and eosin (H&E) staining was performed to analyze the samples.

### 5.12 Immunohistochemistry

For immunohistochemical staining, samples embedded in the OCT compound were cut into 10‐μm‐thick sections at −22°C. To stain the microvessels in the skin tissues, sections were immunofluorescently stained with CD31 (Abcam, ab28364, 1:50) and α‐SMA antibodies (Abcam, ab5694, 1:200). The CD31‐ and α‐SMA‐positive signals were visualized with fluorescein isothiocyanate‐conjugated secondary antibodies (Jackson ImmunoResearch Laboratories, West Grove, PA, USA). These sections were then counterstained with DAPI (Vector Laboratories) and examined using a fluorescence microscope (IX71 inverted microscope; Olympus).

### 5.13 Statistical analysis

All data are presented as mean ± SD. The statistical analysis was performed using GraphPad Prism (GraphPad Software, San Diego, CA, USA). To determine statistical significance, an unpaired Student's *t*‐test was performed to compare two experimental groups, and ordinary one‐way ANOVA was performed for the three experimental groups. Statistical significance was considered when the *p*‐value was less than 0.05 or 0.01.

## AUTHOR CONTRIBUTIONS

**Sung‐Won Kim:** Conceptualization (equal); data curation (equal); investigation (equal); methodology (equal); validation (equal); visualization (equal); writing – original draft (equal). **Gwang‐Bum Im:** Data curation (equal); methodology (equal); visualization (equal); writing – original draft (equal). **Yeong Hwan Kim:** Data curation (equal); investigation (equal). **Suk Ho Bhang:** Funding acquisition (equal); supervision (equal); writing – review and editing (equal).

## CONFLICT OF INTEREST

The authors declare no conflict of interest.

### PEER REVIEW

The peer review history for this article is available at https://publons.com/publon/10.1002/btm2.10462.

## Data Availability

Data available on request from the authors.
